# Exploring entry pathways of microorganisms into an anatomical dissection course

**DOI:** 10.1038/s41598-025-30667-1

**Published:** 2025-12-02

**Authors:** Sebastian Streich, Ruth Ladurner, Sebastian M. Grashorn, Jan Liese, Bernhard Hirt, Peter H. Neckel

**Affiliations:** 1 Institute of Clinical Anatomy and Cell Analysis, Eberhard Karls Universität Tübingen, Österbergstraße 3, 72074 Tübingen, Germany; 2 Institute of Medical Microbiology and Hygiene, University of Tübingen, Tübingen, Germany

**Keywords:** Anatomical dissection course, Body donor, Microbial contamination, Hygiene protocols, Air supply system, Medical education, Microbiology, Anatomy

## Abstract

Anatomical dissection courses are central to medical education. Despite advances in fixation techniques, cadavers remain vulnerable to microbial contamination. Thus, we aimed to assess microbial entry pathways and discuss mitigation strategies in a dissection course setting. Microbial load assessments were performed using sedimentation and contact plate sampling at various points, including doorknobs, gown sleeves, and air inside the dissection hall. Airborne microbial loads were significantly lower under laminar airflow systems compared to areas without controlled air supply, particularly near sinks and entryways (median 17.0 vs. 51.0 CFUs/4 h). The airborne microbial load was significantly lower during unoccupied times compared to dissection hours (median 2.5 vs. 44.0 CFUs/4 h). The dissection hall doorknob showed increased microbial counts after use on 4 of 6 days, though levels remained below critical thresholds. Gown sleeves had a variable but overall low microbial load. Our study suggests that controlled air supply is a key factor for reduced microbial loads. In contrast, the risk of microbial transmission via the doorknobs is negligible. The microbial load on gown sleeves was comparable to that of clean hospital laundry. Future studies should monitor microbial loads on cadaver surfaces to clarify contamination dynamics and refine hygiene protocols.

**Supplementary Information:**

The online version contains supplementary material available at 10.1038/s41598-025-30667-1.

## Introduction

The anatomical dissection course stands as a pivotal component within medical education, offering students invaluable hands-on experience in understanding human anatomy. However, cadaveric dissection also presents potential health and safety risks, coming from the body donor itself, from the fixation used, or from exogenous contamination of the bodies during cadaver handling, storage and dissection. While a chemical fixation process should—under normal circumstances—eliminate all present microorganisms present within and on the surfaces of the cadaver, the contamination and colonization with microorganisms from external sources during storage, handling and dissection represent a real risk.

Despite advances in fixation techniques, cadaveric specimens can harbor a variety of microorganisms, including bacteria, fungi, and viruses^1–7^. This microbial load not only poses potential risks to students and faculty but may also impact the long-term stability of the bodies, which is necessary for a dissection course of up to several months. In the worst case, a contamination and colonization could lead to a microbial overgrowth of the body^8,9^, potentially resulting in a complete loss of the anatomical specimen for teaching purposes and the need for immediate cremation.

Over the past decades, several fixation and storage protocols for anatomical specimens have been published, often alleging advances in fixation and disinfection means. However, cases of excessive microbial growth have also been reported using these protocols, at least partially questioning the claimed benefits of the protocols under student course conditions with the potential of microbial contamination during dissection^8^.

Hardly any systematic studies on the microbial load of an anatomical dissection course have been published^10^. Despite some case reports in the literature and inherited knowledge about hygiene measures in cadaver handling, it is still unclear which of the various procedures and countermeasures during cadaver handling, storage and dissection lead to an actual benefit in protection against microbial entry.

Therefore, we designed this prospective study to assess the entry pathways of microbial contamination into the dissection course at the Department of Anatomy of the Eberhard Karls Universität Tübingen, Germany, using a longitudinal study design throughout the entire course. We aimed to identify potential routes of microbial entry and assessed possibilities to reduce the microbial load. Thus, our study provides pioneering data necessary for the development of evidence-based procedures in cadaver preservation and handling in anatomy, thereby contributing to a reduction of health risks to students and faculty.

## Results

### Risk assessment and study design

**Fig. 1:**
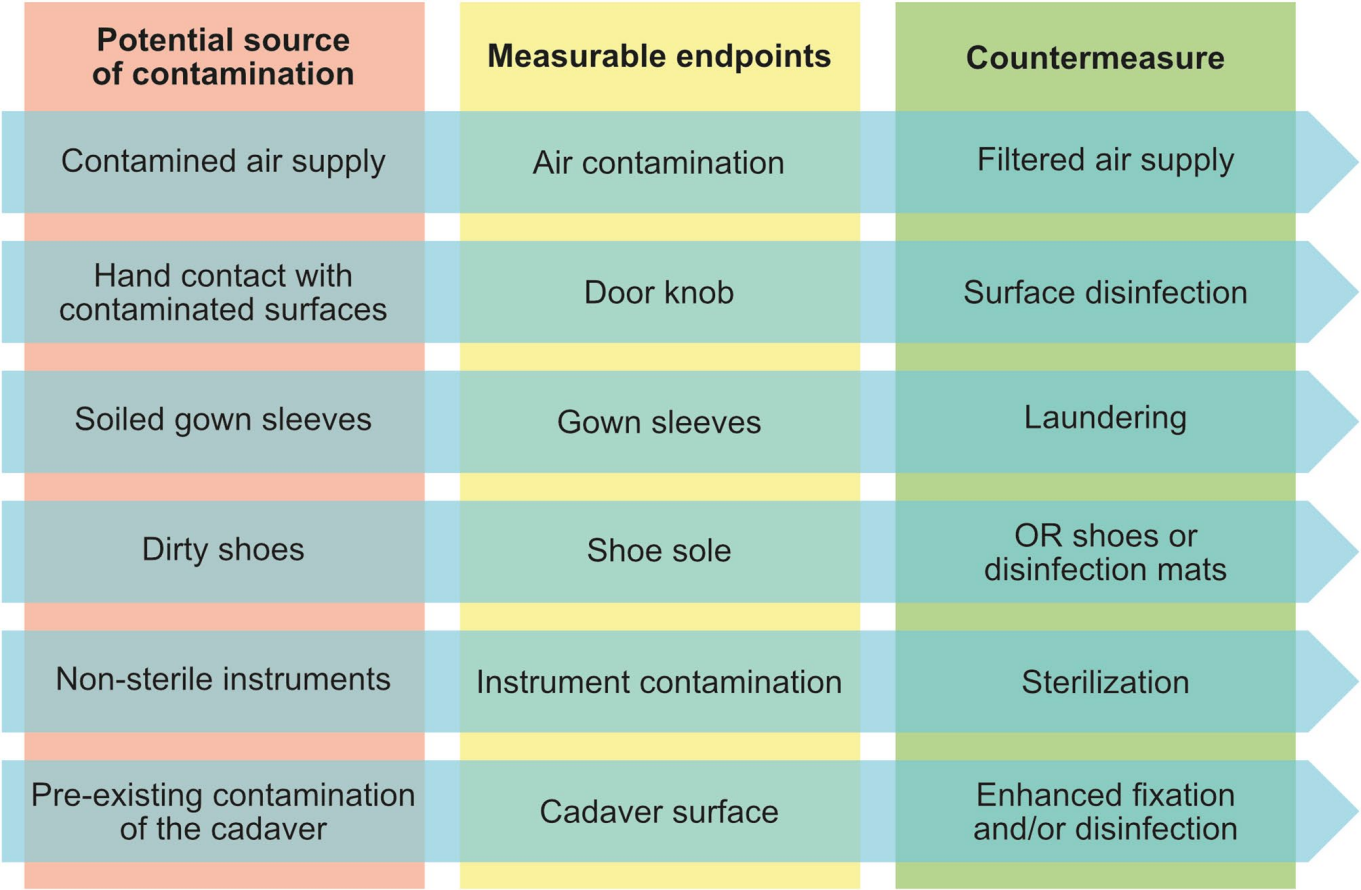
Risk assessment. Shown are potential sources of contamination (red column), measurable endpoints to assess the related microbial burden (yellow column), and possible countermeasures to the contamination sources (green column).

First up, we carried out a narrative risk assessment to identify potential sources of contamination related to the dissection course. In addition to the importance of an entry path, the risk assessment should also define measuring parameters and potential countermeasures. This led to a total of six potential sources of transmission (Fig. 1). The results of the risk assessment along with the sampling analyses are detailed below for the respective entry pathways. In this study, we focused on the first three potential sources, assuming that these are the most relevant, easily measurable and counteractable aspects. Although cadavers can inherently harbor various microorganisms (see above), this aspect was intentionally excluded from the study design, which focused specifically on the entry pathways of exogenous microorganisms.

### Sample acquisition

To monitor and evaluate the microbial load in the dissection course, we acquired samples at eight days, distributed over the entire dissection course. Sedimentation plate samples were taken on three dissection tables, as well as contact plate samples of the gown sleeves of three students and of the doorknob of the dissection hall, totaling in 33 RODAC plate samples and 48 sedimentation plate samples. A schedule of the dissection course and a site plan of the dissection facilities are shown in Supplementary Figure S1 and Supplementary Table S1.

**Fig. 2:**
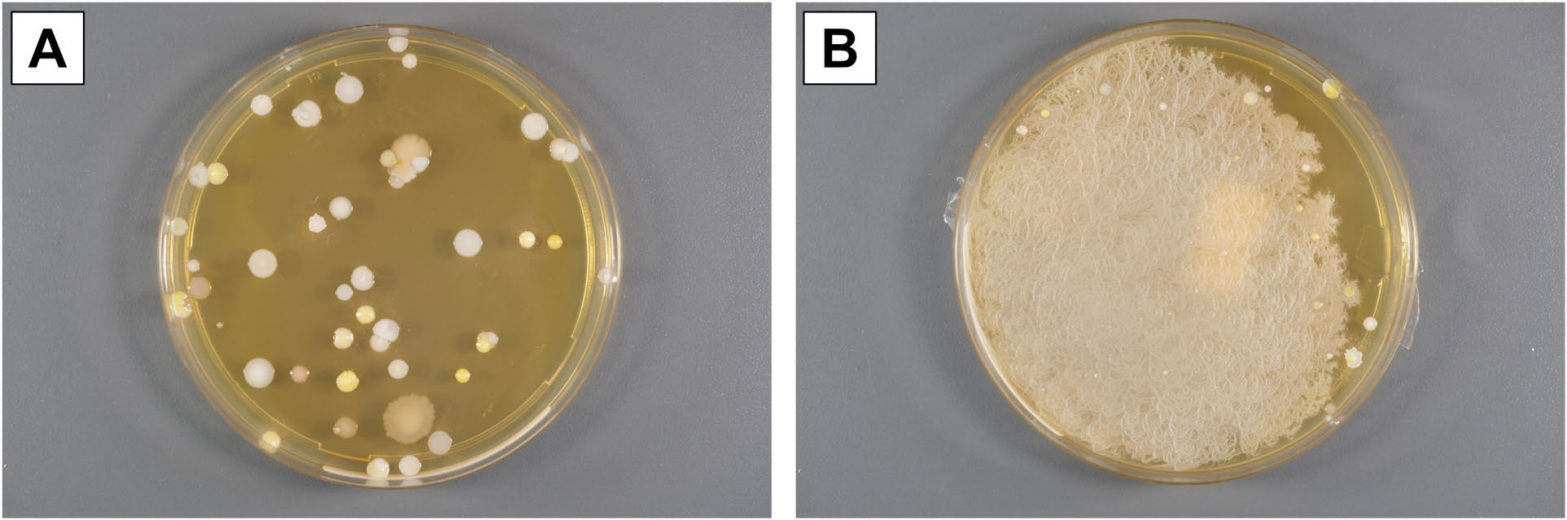
Exemplary findings from sedimentation plates. (**A**) Separate colony forming units of coagulase-negative staphylococci, micrococci, and aerobic spore formers. (**B**) Growth of a bacterial lawn of aerobic spore formers.

The plates were evaluated quantitatively according to the microbiological-infectiological quality standards 22 and 23 (MIQ 22 and MIQ 23) published by the German Society for Hygiene and Microbiology (DGHM)^11,12^, counting the colony-forming units (CFUs) of coagulase-negative staphylococci (CNS), molds (not further differentiated; MND), aerobic spore-forming bacteria (ASF), and micrococci (MIC). Exemplary images of sedimentation plates with growth of different microorganisms are shown in Fig. 2.

### Contaminated air supply leading to potential airborne microbial load

A very important component of a dissecting room, even if it is implemented very differently at various institutes, is the ventilation system^13^. This is usually operated due to the occupational exposure limit of formaldehyde or other carcinogens or toxins in the air, and/or due to odour of the cadavers. At the same time, however, the ventilation system can be used to allow purified air to flow over the specimens or, if not properly maintained, to cause microbial contamination. While most newer institutional buildings are equipped with such ventilation systems, there are still some institutes using open windows as a ventilation method^14^. Depending on the local prerequisites, improvements in this field can be as easy as changing the filters of the ventilation system, or as difficult as constructing a whole new building.

**Fig. 3:**
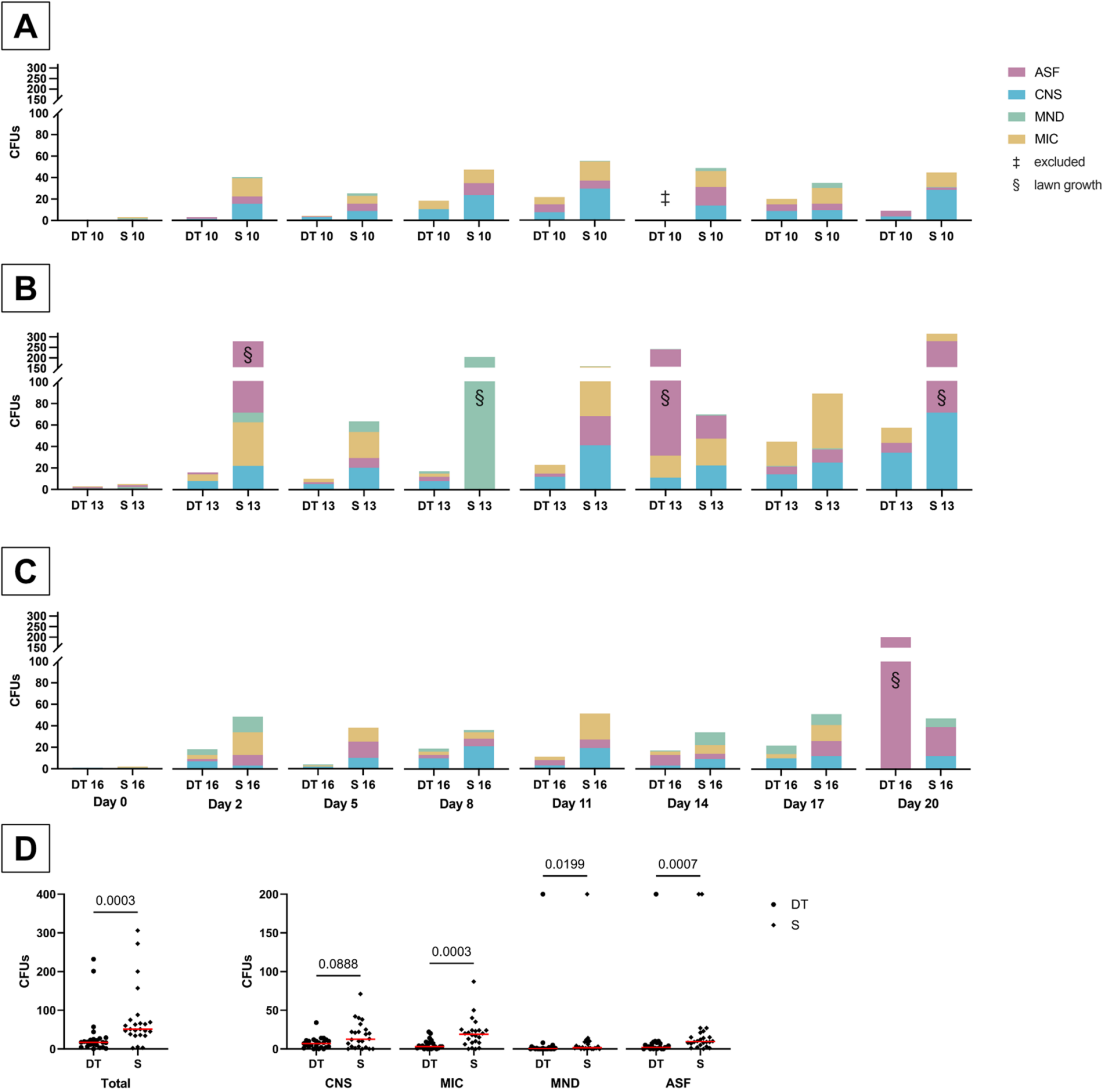
Airborne microbial load after exposure of the sedimentation plates for 4 h, at dissection table 10 (**A**), dissection table 13 (**B**) and dissection table 16 (**C**) and the sills next to the dissection tables, as well as a comparison between dissection tables and lateral sills (**D**). On day 14, one sample (‡) had to be excluded due to touching the settle plate by hand by one of the students. A lawn growth (§) was observed at a total of five samples. The lateral sills exhibited a significantly higher airborne microbial load compared to the dissection tables when totaling all microorganisms (*p* = 0.0003), as well as for micrococci (*p* = 0.0003), molds (*p* = 0.0199) and aerobic spore formers (*p* = 0.0007). Median indicated as a red bar. The original numeric data of this figure can also be found in Supplementary Table S2. DT: Dissection table. S: Sill next to the dissection table. CFUs: colony forming units. CNS: coagulase-negative staphylococci. *MIC* micrococci, *MND *molds (not differentiated), *ASF *aerobic spore formers.

Measuring the microbial air pollution, a total of 48 sedimentation plate samples with an exposure time of 4 h each were taken at tables 10, 13 and 16 (Fig. 3A–C). However, one of those samples from dissection table 10 had to be excluded due to accidently touching the plate by one of the students. Additionally, 48 sedimentation plate samples with an exposure time of 1 h each were taken as an extra control and served to evaluate short-term microbial load and kinetics. It is noteworthy that the reference values in the GMP guideline annex 1, Sect. 4.31^15^ are based on 4 h values, therefore the following results focus on those values.

The microbial load in the air was systematically assessed at multiple sites, including the lateral shelves and dissection tables, the latter of which are positioned under a laminar airflow system (see Materials and methods section for details on the layout of the dissection hall). Interestingly, the microbial load on the lateral shelves consistently exceeded that on the dissection tables, except for two samples. Taking all microorganisms of an individual plate in total, the sedimentation plates of the lateral sill exhibited a significantly higher microbial load than the plates of the dissection tables (median 17.0 vs. 51.0 CFUs/4 h, *p* = 0.0003, n_dissection table_ = 23, n_sill_ = 24, two-sided Mann-Whitney U test). Commonly identified microorganisms included coagulase-negative staphylococci, micrococci, and aerobic spore-formers, which were abundantly present in both the air over the dissection tables and on the lateral shelves. Especially micrococci (median 3.0 vs. 19.0 CFUs/4 h, *p* = 0.0003, n_dissection table_ = 23, n_sill_ = 24, two-sided Mann-Whitney U test) and aerobic spore-formers (median 2.0 vs. 9.5 CFUs/4 h, *p* = 0.0007, n_dissection table_ = 23, n_sill_ = 24, two-sided Mann-Whitney U test) showed a significantly higher presence in the air over the lateral sill compared to the dissection table (see also Fig. 3D and Supplementary Table S2).

Beside the samples taken directly in the course, control samples were taken prior to the first course day. Those samples revealed a maximum number of 3 CFUs/4 hours at the dissection table, and 5 CFUs/4 hours at the sill. The combined microbial contamination (dissection tables and sills) of the air in the dissection hall was notably lower when the room was unoccupied, compared to days when students were present (median 2.5 vs. 44.0 CFUs/4 h, *p* < 0.0001, n_unoccupied_ = 6, n_occupied_ = 41, one-sided Mann-Whitney U test). The separate analysis of the microbial load of dissection tables (median 1.0 vs. 18.5 CFUs/4 h, *p* = 0.0006, n_unoccupied_ = 3, n_occupied_ = 20, one-sided Mann-Whitney U test) and lateral shelves (median 3.0 vs. 60.0 CFUs/4 h, *p* = 0.0005, n_unoccupied_ = 3, n_occupied_ = 21, one-sided Mann-Whitney U test) produced corresponding results. Interestingly, we found that the airborne microbial load of the dissection tables and the lateral sill tended to increase throughout the dissection course, however, without reaching a statistically significant level (*p* > 0.13 and *p* > 0.20, respectively, Kruskal–Wallis H test with Dunn’s multiple comparisons test).

In the samples of the dissection tables, fungal contamination was sporadically observed with low CFU numbers compared to the other species, apart from the lateral sills, where mold was frequently detected. Notably, a dense overgrowth with molds on an agar plate from the lateral shelf occurred on only one occasion.

Moreover, the samples from the lateral sill at dissection table 13, located near a sink, demonstrated lawn-like microbial growth in three out of seven measurements when the room was occupied. This phenomenon was in stark contrast to the other sampling sites, where such extensive growth was observed only twice, on one agar plate each from dissection tables 13 and 16.

The results of the 1-hour sedimentation plate samples are shown in Supplementary Table S3 and Supplementary Figure S2 and are discussed below.

### Hand contact with contaminated surfaces, especially the doorknob

**Fig. 4:**
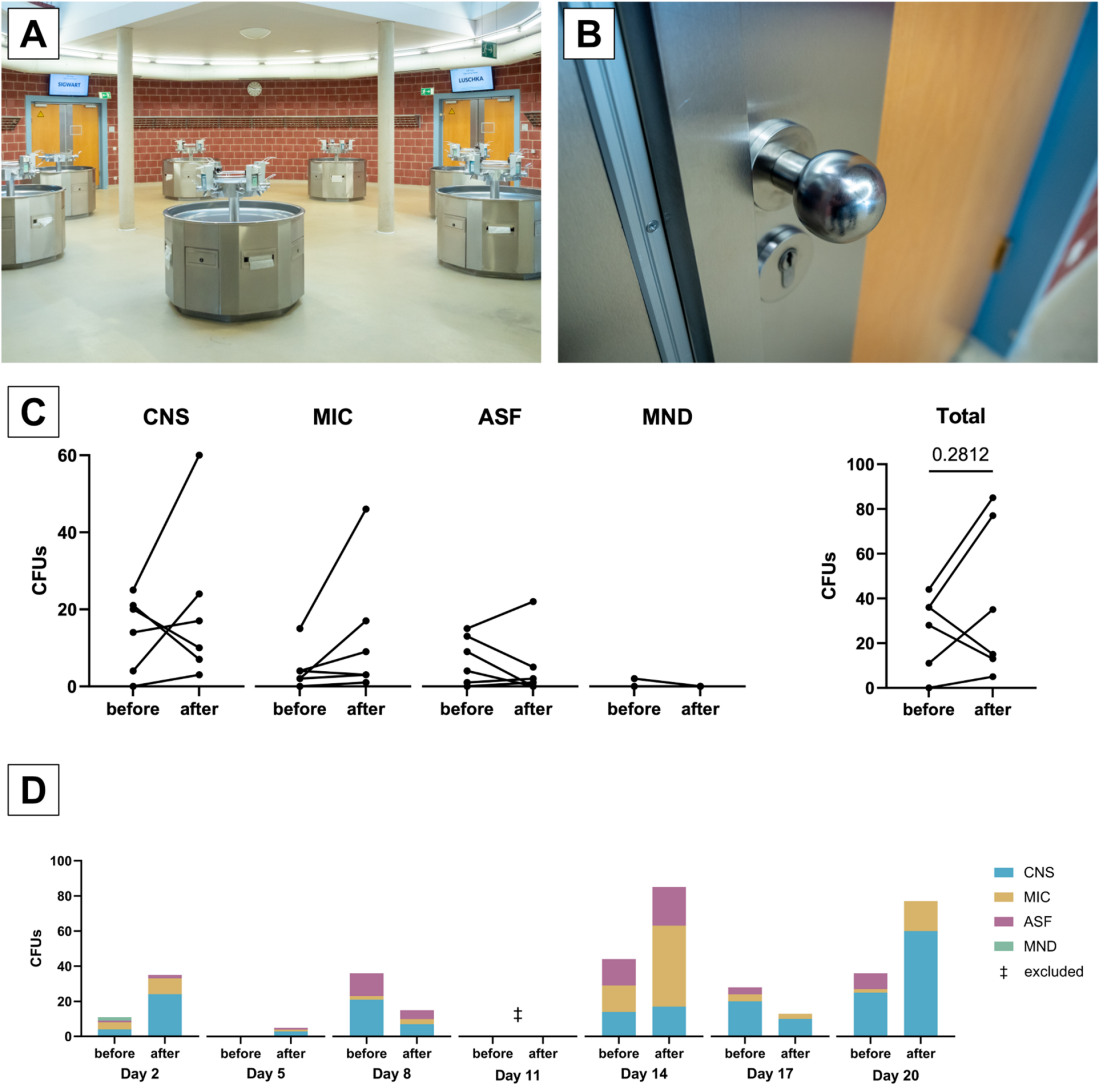
Microbial load of the dissection halls doorknob. (**A**) Vestibule of the dissection hall with sinks and coat hooks, providing access to the dissection hall through two doors. (**B**) Doorknob of one of the doors of the dissection hall. (**C**) Changes in microbial load of the doorknob by groups of microorganisms and in total across the measurement time (before and after the course). (**D**) Microbial load of the doorknob, plotted by course day and time of measurement. Course day 11 had to be excluded (‡) due to a communication error with the microbiological lab. The original numeric data of this figure can also be found in Supplementary Table S4. CFU: colony forming units. CNS: coagulase-negative staphylococci. *MIC *micrococci, *MND *molds (not differentiated), *ASF *aerobic spore formers.

After the students had put on their gowns in the washroom (Fig. 4A), they washed and/or disinfected their hands. Some of the students then put on non-sterile gloves, with which they worked on the cadavers later on. Other students put on the gloves in the dissection hall. As a result, some students touched the doorknob (Fig. 4B) to the dissection hall with gloves, some without gloves, of the latter only partially with disinfected hands. This allowed skin microorganisms to get onto the surface of the gloves, which were then used to work on the specimen. To measure the possible spread of microorganisms via the doorknob, contact plate samples were a suitable monitoring tool. Regular surface disinfection of the doorknob could be a simple countermeasure.

The microbial load on the outer doorknob of the dissection hall was measured using contact plates. Sampling was conducted twice per course day: once before the start of the course (before students entered the building), and once after the course had ended.

On four out of the six sampled days, the microbial load on the door handle was higher after the course than before (Fig. 4C). Conversely, on the remaining two days, a decrease in microbial load was observed after the course. Overall, the difference of the microbial load of the doorknob before and after the course days was not significant (median 32.0 vs. 25.0 CFUs, *p* = 0.2812, n_before_ = 6, n_after_ = 6, two-sided Wilcoxon signed rank test). The dominant microorganisms detected included coagulase-negative staphylococci, aerobic spore-formers, and micrococci, whereas molds were found only sporadically and in low colony-forming unit counts. The results of the doorknob sampling are also shown in Fig. 4, Supplementary Table S4 and Supplementary Table S5.

CNS, ASF and MIC were observed on all days, while MND were only detected on day 2 with 2 CFUs. CNS accounted for 205/385 CFUs (53,2%), MIC for 106/385 CFUs (27,5%), ASF for 72/385 CFUs (18,7%), and MND for 2/385 CFUs (0,5%). On most days, the majority of CFUs originated from the CNS group. However, on day 14, MIC and ASF were more abundant than CNS.

CNS shows an increase over dissection course time, with notable spikes on days 2 and 20 (Fig. 4D). Intriguingly, the CFU count was less after the course than before on days 8 and 17. MIC also increased over time but showed a peak on day 14.

### Soiled gown sleeves

The students wore their normal streetwear for the dissection and put on a white cotton gown. They had to procure these gowns themselves and wash them on their own throughout the course. However, some students irregularly used the hospital’s pool laundry and picked up a new gown before each course day, which they returned to the hospital laundry after the course day. Although the students were required to launder their gowns regularly, the washing of the gowns was not monitored. As a result, some students attended the dissection course with visibly soiled gown sleeves. Contact plate samples were taken to measure the microbial load on the gown sleeves. One way of minimizing the potential transmission of microorganisms could be to ensure that the gowns are cleaned regularly and sufficiently.

To assess the microbial contamination on gown sleeves, contact plate samples were collected from the outer surface of the gown cuff worn by students at three dissection tables over seven measurement days. On each occasion, a single student’s lab coat sleeve was sampled at each table. The students were selected randomly; thus, it was possible that the same gown was sampled on several occasions. Due to this variability in sampling, no conclusive statements can be made regarding the temporal progression of microbial contamination on individual lab gown sleeves.

During the sampling process, some gowns exhibited noticeable visual soiling. However, this observation was not systematically recorded and was not used as a criterion for including or excluding samples from the study. As a result, the potential influence of visible contamination on microbial load was not analyzed in this study.

**Fig. 5:**
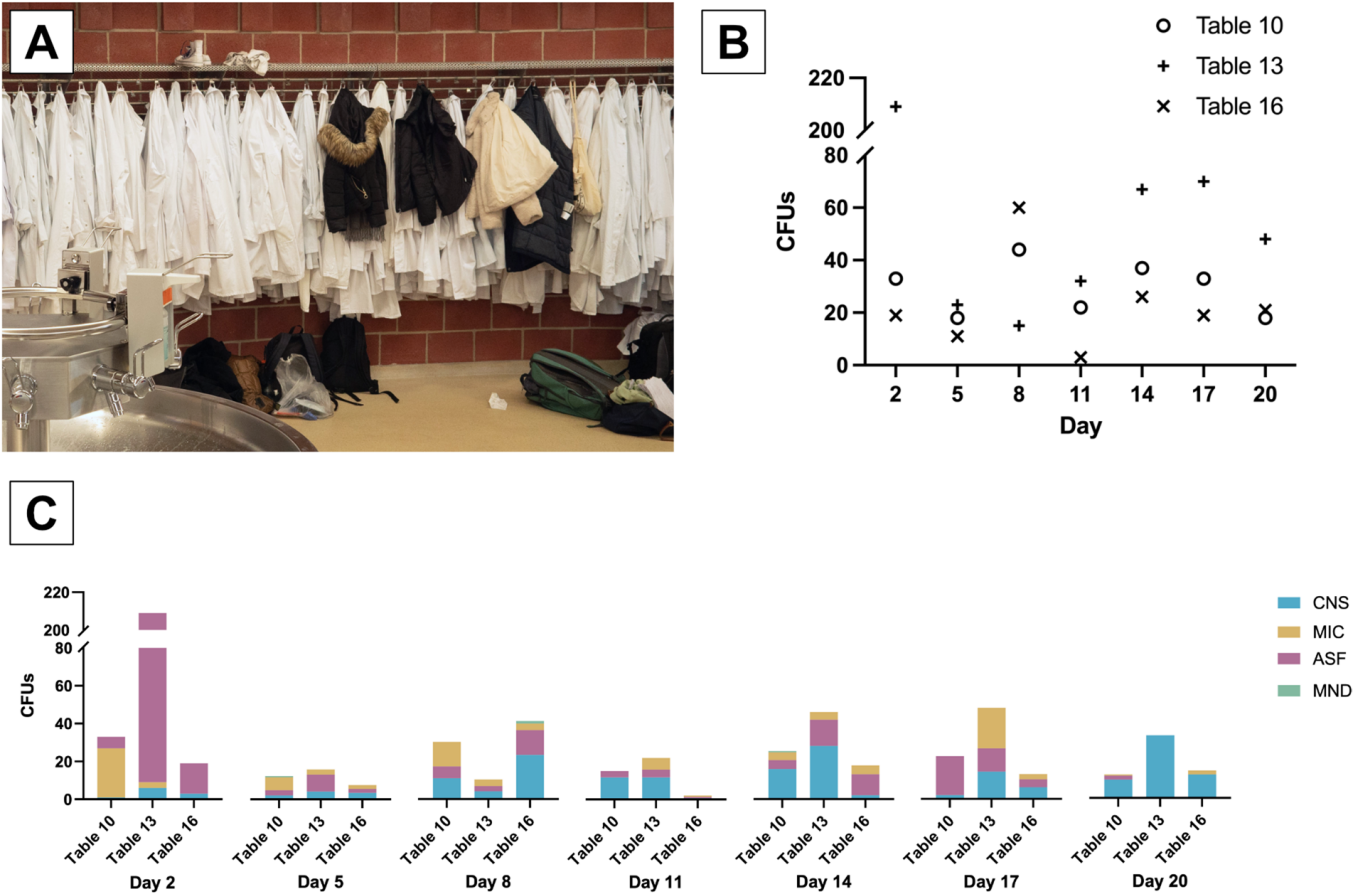
Microbial load of the gown sleeves. (**A**) Gowns for dissection and normal coats hanging on the same coat hooks in the vestibule, creating a potential for transmission of microorganisms. (**B**) Totaled microbial load of gown sleeves from three randomly selected students from dissection tables 10, 13 and 16. (**C**) Microbial load of the gown sleeves, differentiated across species. The original numeric data of this figure can also be found in Supplementary Table S6. CFUs: colony forming units. CNS: coagulase-negative staphylococci. *MIC *micrococci, *MND *molds (not differentiated), *ASF *aerobic spore formers.

In total, 21 samples (7 sampling days with 3 students each) of students’ gown sleeves were taken. Altogether, 771 CFUs were detected, resulting in a mean load of 36,7 CFUs per gown sleeve and day. However, the distribution of the number of CFUs across the different gowns and days was highly variable, ranging from 3 to 209 CFUs (including one case with lawn growth, considered as CFU count of 200, see Materials and methods section for details). Moreover, the distribution of the microorganisms detected was heterogeneous: In total, 377 CFUs ASF, 260 CFUs CNS, 130 CFUs MIC and 4 CFUs MND were detected (see also Fig. 5 and Supplementary Table S6).

## Discussion

Anatomical dissection courses are still one of the core components of pre-clinical medical education. In recent years, dissection courses have come under increasing pressure and justification due to health risks such as potential chemical hazards from formaldehyde or other fixing substances or biological hazards such as microbial contamination and colonization of the cadavers. Therefore, we investigated the microbial load and corresponding entry pathways of microbial contamination into an anatomical dissection course, aiming to identify potentially counteractable sources of contamination.

In the preceding risk assessment, five potential sources of microbial contamination were identified.

### Doorknob as a central contact point, but with limited influence on microbial contamination

The microbial load of the doorknob of the door in between the vestibule and the dissection hall was higher after the course than before on four out of six sampling days, indicating significant exposure of the doorknob to students’ hand contact during dissection hours. On each course day, around 360 medical students attended the dissection course. Yet, a precise number of hand contacts with the doorknob is impossible to estimate since we did not monitor the number of entries per students nor the handling (e.g., single students holding the door open for their peers).

In most of the sampling days, we found a higher CFU count after the dissection course than before. Counterintuitively, the microbial load was lower after the course on two days (dissection day 8 and 17). Since the cleaning routine of the dissection hall, its vestibule and of the doorknobs remained constant across the complete dissection course, we can rule out the intensity of cleaning as a confounding factor to this finding. However, as the students washed and disinfected their hands prior to entering the dissection hall, we consider it conceivable that students touched the doorknob with their hands still wet with disinfectant thereby accidentally cleaning the doorknob, resulting in lower microbial load after the course.

In fact, our results on the microbial load of the doorknob are in line with the data from studies carried out inside and outside of healthcare facilities^16–18^. Moreover, our strict institutional guidelines (Institute of Medical Microbiology and Hygiene, University of Tübingen) for *disinfected* surfaces in healthcare facilities consider a score of 25 to 100 CFUs per contact plate as “critical”, however not “in need of action” (CFU count above 100). CFU counts between 5 and 25 are considered “acceptable”. Since all measurements of the (*not* disinfected) doorknob exhibited CFU values below 100 (5 out of 12 samples even below 25 CFUs), the doorknob is astonishingly clean and not more of a source of contamination than any other surface that can be touched by a student during the dissection course.

### Gown sleeves show remarkably low CFU counts overall

The students’ gown sleeves reflected a diverse and variable microbial load. Across seven sampling days, 771 CFUs were detected, with an average of 36.7 CFUs per gown sleeve per day. The variability in CFU counts, ranging from 3 to 209, suggests that gown sleeves are exposed to different levels of contamination depending on individual activity, environmental factors, and possibly inconsistent cleaning practices. The notably high CFU count of 209 on day 2 at dissection table 13 was attributed to bacterial lawn growth of ASF, indicating isolated but significant contamination events.

The distribution of microorganisms on gown sleeves mirrored patterns observed on the doorknob. ASF accounted for the highest proportion of detected CFUs (377 out of 771), followed by CNS (260 CFUs), MIC (130 CFUs), and minimal MND (4 CFUs). This heterogeneous distribution highlights that microbial contamination varies by dissection table and individual contact, reinforcing the need for consistent hygiene protocols and gown laundering to prevent cross-contamination. This is particularly necessary given the close contact between (a) the coats and/or (b) the coats and the normal street clothes on the wardrobe, which could lead to significant cross-contamination.

Interestingly, visual soiling of gowns was observed during sampling but not systematically recorded, so future studies should incorporate this factor to evaluate its potential correlation with microbial load. The absence of this data limits the ability to draw conclusive links between visible contamination and CFU counts.

While the students were encouraged to wash their gowns on a regular basis, the actual cleaning intervals were not monitored systematically. Therefore, a clear statement on the effect of regular cleaning of the gowns cannot be made.

Moreover, the microbial load of students’ gown sleeves averaged at 36.7 CFUs and it is therefore comparable to the standardized cleaned hospital laundry of the University Hospital Tübingen with up to 28 CFUs per sample. Therefore, a substantial microbial contamination of the cadavers via soiled gown sleeves seems less likely. Nevertheless, to unravel the significance of gown sleeves as a vector for cadaver contamination, a direct monitoring of the sleeves as well as the cadavers’ surface identifying microbial species is required in future studies.

### Filtered air supply as a key factor for reduced airborne microbial load

A key part of this study was the assessment of the airborne microbial load inside of the dissection hall. Consistent with expectations, the laminar airflow system above the dissection tables effectively reduced microbial contamination compared to lateral sills. This aligns with previous studies emphasizing the efficacy of (laminar) airflow systems in minimizing microbial dispersion in controlled environments^19–21^.

The consistently higher microbial counts on the lateral sills, as opposed to the dissection tables, suggest inadequate air circulation in these peripheral areas. Interestingly, samples from the lateral sills near the sink exhibited exceptional microbial growth, particularly near to dissection table 13. The proximity to the sink – a potential source of microbial-contaminated aerosols – likely contributed to this finding. Furthermore, the nearby door poses a possible source of unfiltered and therefore contaminated air. Table 13 and especially the sink nearby also have the largest number of people walking by: All students entering or leaving the dissection hall have to pass this area. These findings underline the importance of a controlled and filtered air supply as well as the influence of human behavior on the airborne microbial load.

This is further substantiated by the significantly lower CFU counts during unoccupied periods (dissection day 0) compared to those measured during active use of the dissection hall. Additionally, the results from the short-term sampling (1 h exposure time of sedimentation plates, see Supplementary Figure S2 and Supplementary Table S3) indicate that (a) working on a cadaver – even for a short time – considerably increases the microbial load, and (b) course periods with higher activity (i.e., exchange of student cohorts and cleaning of used instruments) have only a limited influence on the overall airborne microbial burden. However, our experimental setup does not resolve whether the sheer presence of people and the subsequently disturbed laminar airflow as described by Dahncke et al.^22^, or a potentially added microbial load with germs from the mouth-nose-throat area lead to higher CFU counts during active dissection.

While a direct comparison of the data is not possible due to a different measurement method of the airborne microbial load, Keiler et al. suggest that the increased microbial load during occupation of the dissection hall originates from ambient air entry^10^. In our case, the doors connecting the vestibule to the dissection hall represent the largest possible entry sides of ambient air; however, our study lacks data on the airborne microbial load inside the vestibule, so a statement on the effect of this potential entry pathway is not possible. Furthermore, other studies have shown that the laminar aspect of a laminar flow system is dramatically decreased in the presence of multiple people^22,23^. Taken altogether, the effect of a *laminar* air supply on the microbial load is at least questionable, while *filtered* and *controlled* air supply is a key factor for the reduction of the airborne microbial load.

### Dirty shoes and non-sterile instruments could be important for future studies

In addition to wearing street clothes, the students also wore their normal street shoes in the dissection hall. This allows soil and earth microorganisms to enter the dissection hall’s floor, and studies have shown potential airborne transmission of floor microorganisms by re-aerosolization due to air movement or human activities^24^. One possible countermeasure would be the introduction of daily cleaned operating room (OR) shoes, which might not be logistically feasible depending on the number of participants. As an alternative to this approach, it would be also possible to implement disinfection mats at the entrance to the dissection hall. However, we considered that the airborne spread of microorganisms from the floor to the cadaver is highly unlikely in our specific case, as the air supply provides a filtered, downward-oriented airflow directly over the cadaver’s surface, while waste air is extracted near the floor. In other institutions without comparable air supply systems, this aspect should be considered as a potential transmission route.

While the reprocessing and sterilization of surgical instruments for use on patients is standard, a corresponding procedure for anatomical dissecting instruments has not yet been established, mainly due to logistical and financial limitations during an anatomical dissection course. Even though non-sterilized instruments are a potential source of microbial contamination, appropriate countermeasures do not seem reasonable in the context of anatomy.

Overall, both the soiled shoes and the non-sterile instruments were considered as less important for the aim of this study, which was to identify potentially counteractable contamination sources. However, these points should be addressed in future studies.

## Conclusion

While partially exceptionally high CFU counts were detected in this study, none of the cadavers of the dissection tables monitored showed visible microbial growth, and therefore none of these cadavers had to be taken out of the dissection course. However, this study lacks a direct monitoring of the microbial load on the cadavers’ surface, or the linen sheets used to cover the cadavers, which should be addressed in future studies. While hygiene guidelines for students and staff are important, our study highlights the importance of a controlled and filtered air supply. Moreover, our results provide pioneering evidence to substantiate or refute inherited assumptions of microbial contamination routes in anatomical dissection courses.

## Materials and methods

### Body donors

The dissection course for medical students included 18 human cadavers (9 male : 9 female) donated to the Institute of Clinical Anatomy and Cell Analysis in Tübingen by female and male volunteers aged between 72 and 94 years. The body donors gave their informed consent in concert with the declaration of Helsinki to use their cadavers for teaching and research purposes. The procedure was approved by the local ethical authorities (project no. 237/2007BO1). The sex, age, body weight, body height, postmortal interval, storage after fixation, and cause of death are summarized in Supplementary Table S7.

### Fixation and storage of body donors

The fixation was carried out by intravasal infusion via the femoral artery using an IJT-50 injection system (Thalheimer, Ellwangen, Germany). Depending on the condition of the cadaver’s vascular system, we used perfusion pressure of 0.5 to 1.0 bar. The volume of the fixation solution was 15 to 20 L, depending on the body weight of the donor. The fixation solution was based on the recipe by Tutsch^25^ and consisted of 45.5% (v/v) ethanol (Carl Roth GmbH + Co. KG, Karlsruhe, Germany), 23.5% (v/v) glycerol (Roth), 2% (v/v) formaldehyde (Roth), and 3.6% (v/v) Lysoformin (Lysoform – Dr. Hans Rosemann GmbH, Berlin, Germany) in water. The product “Lysoformin”, which was used, contained formaldehyde, glutaraldehyde, sodium alkyl ether sulphate and ethanol; it must not be confused with the renewed product lineup of the Lysoformin series, containing quaternary ammonium compounds. In some of the cadavers, Lysoformin had to be replaced by 0.7% (v/v) Aldasan 2000 (Lysoform – Dr. Hans Rosemann GmbH) due to the change of the composition of Lysoformin. Afterwards, the cadavers were wrapped in wet linen and air-tight polyethylene foil, and kept at 19 °C.

### Design of the dissection course

The dissection course took place from October 17th to December 18th, 2023. 361 medical students of the 2nd and 3rd semester divided in two cohorts (morning and afternoon) carried out supervised dissections on 18 body donors (i.e., 2 × 10 students / body donor) on 21 days stretched over the entire course period of two and a half months (see course schedule in Supplementary Table S1). At each dissection table, a student tutor was present, as well as a lecturer, who was responsible for two to three tables. Each cohort dissected over three hours per day, totaling in a dissection time of six hours per day. However, the first day of the course was only three hours in total. Between both cohorts, the cadavers were wrapped in wet linen. The linen sheets were regularly replaced with freshly hygiene-laundered ones, or if visibly soiled. Moreover, the students had access to the dissection hall to prepare for the exams on four separate occasions, on which no dissections were carried out. Furthermore, oral practical examinations were performed on the cadavers on three days.

### Layout of the dissection hall

The course took place in the dissection hall at the Institute of Clinical Anatomy and Cell Analysis at the University Tübingen, Germany. The dissection hall has a half-ring-shaped layout and a floor space of 316.30 m^2^, of which approximately 80 m^2^ were not used for the medical students dissection course (see Supplementary Figure S1 for a scheme of the dissection hall). Two doors, equipped with automatic door closers, provided access to the dissecting hall; these doors had to be kept closed when not entering or leaving the dissection hall. The doorknobs are of a grip-type and made of polished stainless steel. The windows at the large curvature of the dissection hall were kept closed during and in between course hours. The dissection hall is equipped with a ventilation system, which provides a 19-fold air exchange per hour. The air supply comes from filtered, tempered and humidity controlled outdoor air, with a target temperature of 16 °C. The air filters are serviced and changed once a year, most recently six months before the start of the course. Fresh air is supplied over laminar-flow hoods, which are mounted over each dissection table. Waste air is extracted through vents near the ground (see also Supplementary Figure S3).

### Hygiene regulations

Students participating in the dissection course had to wear a white lab gown made of woven cotton fabric over their normal clothes, and non-sterile nitril gloves, as well as a surgical mask (according to the FFP1 standard). Some students wore an FFP2/N95/KN95 mask on a voluntary basis. The lecturers wore scrubs and surgical gowns, as well as nitril gloves and surgical masks. Prior to the course, the students and the lecturers washed their hands in the vestibule of the dissection hall (see Supplementary Figure S4) with soap and performed a hygienic hand disinfection, using a hand disinfectant with a limited virucidal plus rating. It is noteworthy that we did not monitor if the students carried out hand disinfection correctly.

### Sample acquisition days

The first microbiological samples were acquired one day prior to the first dissection day, when the cadavers were already in the dissection hall, but no students were present yet. After that, samples were taken on every third course day, totaling in seven sample acquisition days throughout the course. The acquisition days are marked with a section sign (§) in the course schedule in Supplementary Table S1.

### Sampling and CFU quantification on contact plates

For surface contamination monitoring, replicate organism detection and counting (RODAC) plates (Thermo Fisher Scientific), containing tryptone soya agar (TSA) with lecithin, histidine and polysorbate 80 for disinhibition of disinfectants, with a surface area of 24 cm^2^ were used. Samples were taken by standard contact plate sampling procedures sampling the outer doorknob of one of the two doors prior to the course and after the course. Furthermore, a gown sleeve of a randomly selected student at dissection tables 10, 13 and 16 was sampled according to DIN EN 14,065^26^. Those students were asked to participate in this study on a voluntary basis and always had the possibility to decline the participation without any disadvantages. Acquisition sites for contact plates are marked with a section sign (§) in Supplementary Figure S1.

The sampled RODAC plates were then transferred to the lab of the Institute of Medical Microbiology and Hygiene and processed according to the microbiological-infectiological quality standards 22 and 23 (MIQ 22 and MIQ 23) of the German Society for Hygiene and Microbiology (DGHM)^11,12^. All samples were incubated for 42 to 48 h at 35–39 °C to determine the bacterial count. Afterwards, a second incubation followed for further 114–120 h at 28–32 °C to determine the fungal count. All grown colonies during this time are counted as colony-forming units (CFUs), and colonies were morphologically differentiated as micrococci (MIC), coagulase-negative staphylococci (CNS), aerobic spore forms (ASF) and molds (MND). Growth of more than or equal to 200 CFUs was classified as lawn growth, and those samples were included in the quantitative analyses with a value 200 CFUs.

### Passive air monitoring in the dissection hall

The air in the dissection hall was monitored using sedimentation plates with tryptic soy agar with lecithin, polysorbate 80, histidin and thiosulfate for disinhibition (TSA + LTHThio) (Merck KGaA), which were exposed for 1 and 4 h each. Those plates had a surface area of 64 cm^2^. Sedimentation plate samples were taken under the laminar flow hood of dissection tables 10, 13 and 16 (directly on top of the cadaver), as well as on the sill next to the dissection table 10/11, 13/14 and 15/16. Acquisition sites for settle plates are marked with a hash (#) in Supplementary Figure S1.

The incubation and evaluation process of the settle plates was identical to that of the RODAC plates.

Reference values for the airborne bacterial count were defined based on the GMP guideline annex 1, Sect. 4.31^15^.

### Photo documentation

Photos of the course setup and the sampling points were taken using a Panasonic LUMIX GH5 mirrorless camera with an Olympus M.Zuiko Digital ED 12–40 mm F2.8 PRO lens.

### Statistical analysis

Statistical significance was concluded for values *p* < 0.05. Significance tests were performed using the Mann–Whitney U test and the Kruskal–Wallis H test (for sedimentation plate samples), as well as the Wilcoxon signed-rank test (for RODAC plate samples of the doorknob). Statistical analyses were carried out using GraphPad Prism, version 10.5.0.

## Supplementary Information

Below is the link to the electronic supplementary material.

Supplementary Material 1

## Data Availability

All data generated or analyzed during this study are included in this article and its Supplementary Information files.
