# Supplementary material for: Exploring entry pathways of microorganisms into an anatomical dissection course
Source: Sci Rep. 2025 Dec 2;15:43022. doi: 10.1038/s41598-025-30667-1 (PMC12675709; doi:10.1038/s41598-025-30667-1)
Supplement: Supplementary file 1 — Supplementary Material 1 [file 41598_2025_30667_MOESM1_ESM.pdf]

Supplementary Material

Exploring Entry Pathways of Microorganisms into an Anatomical Dissection Course

Sebastian Streich, Ruth Ladurner, Sebastian M. Grashorn, Jan Liese, Bernhard Hirt, Peter H. Neckel

Contents

Supplementary Figures 3

Supplementary Tables 7

Supplementary Figures

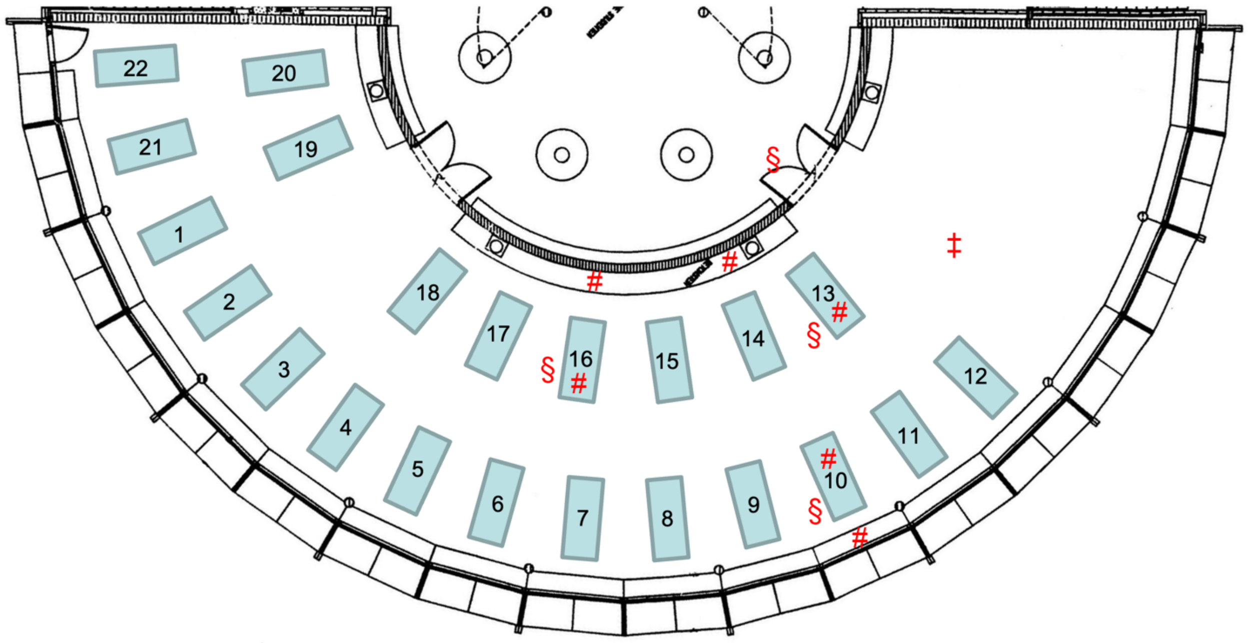

Supplementary Figure S1: Schematic layout of the dissection hall. The vestibule (Supplementary Figure S4) is shown at the top center. Two doors providing access to the dissection hall are located at the small curvature, while a window front is at the large curvature. The light blue rectangles indicate the dissection tables. §: Acquisition sites for contact plate samples. #: Acquisition sites for sedimentation plate samples. ‡: Location of the photograph in Supplementary Figure S3.

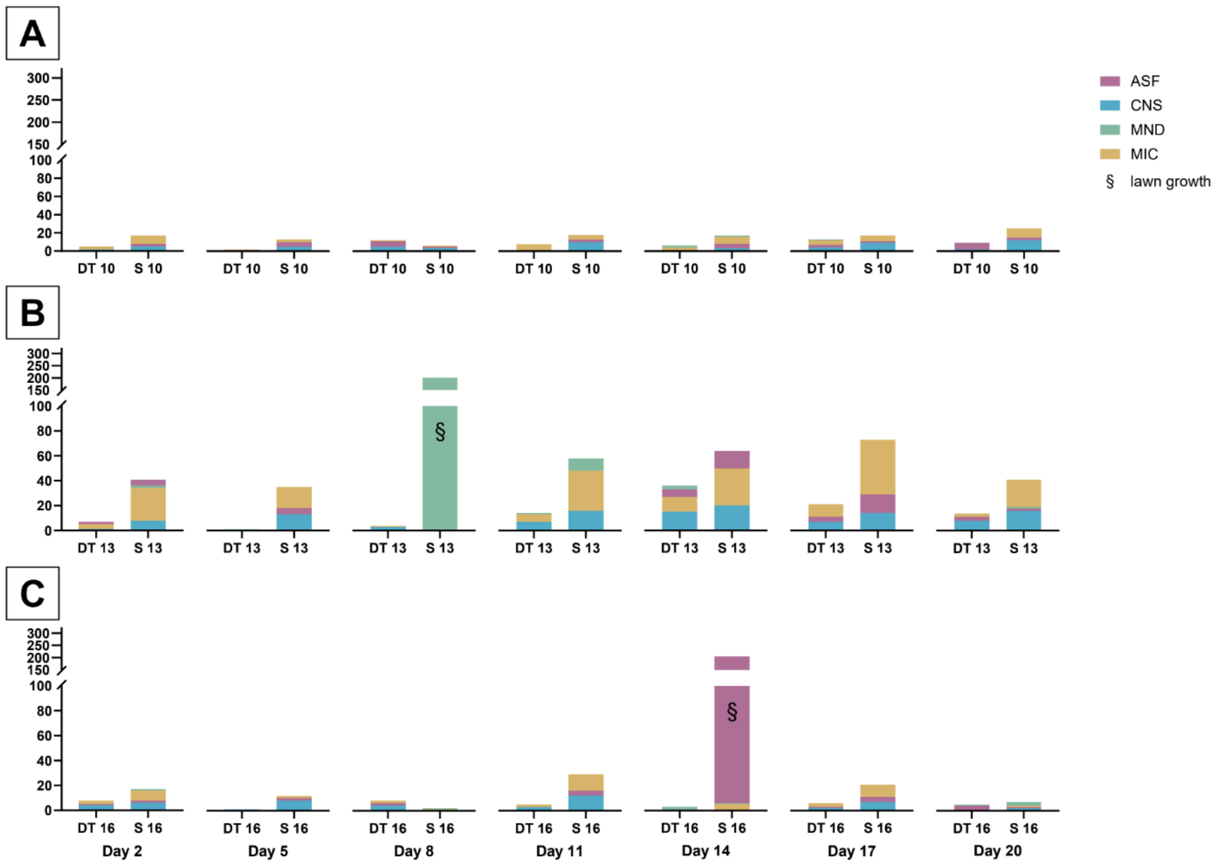

Supplementary Figure S2: Airborne microbial load after exposure of the sedimentation plates for 1 hour, at dissection table 10 (A), dissection table 13 (B) and dissection table 16 (C) and the sills next to the dissection tables. A lawn growth (§) was observed at a total of two samples. DT: Dissection table. S: Sill next to the dissection table. CFUs: colony forming units. CNS: coagulase-negative staphylococci. MIC: micrococci. MND: molds (not differentiated). ASF: aerobic spore formers.

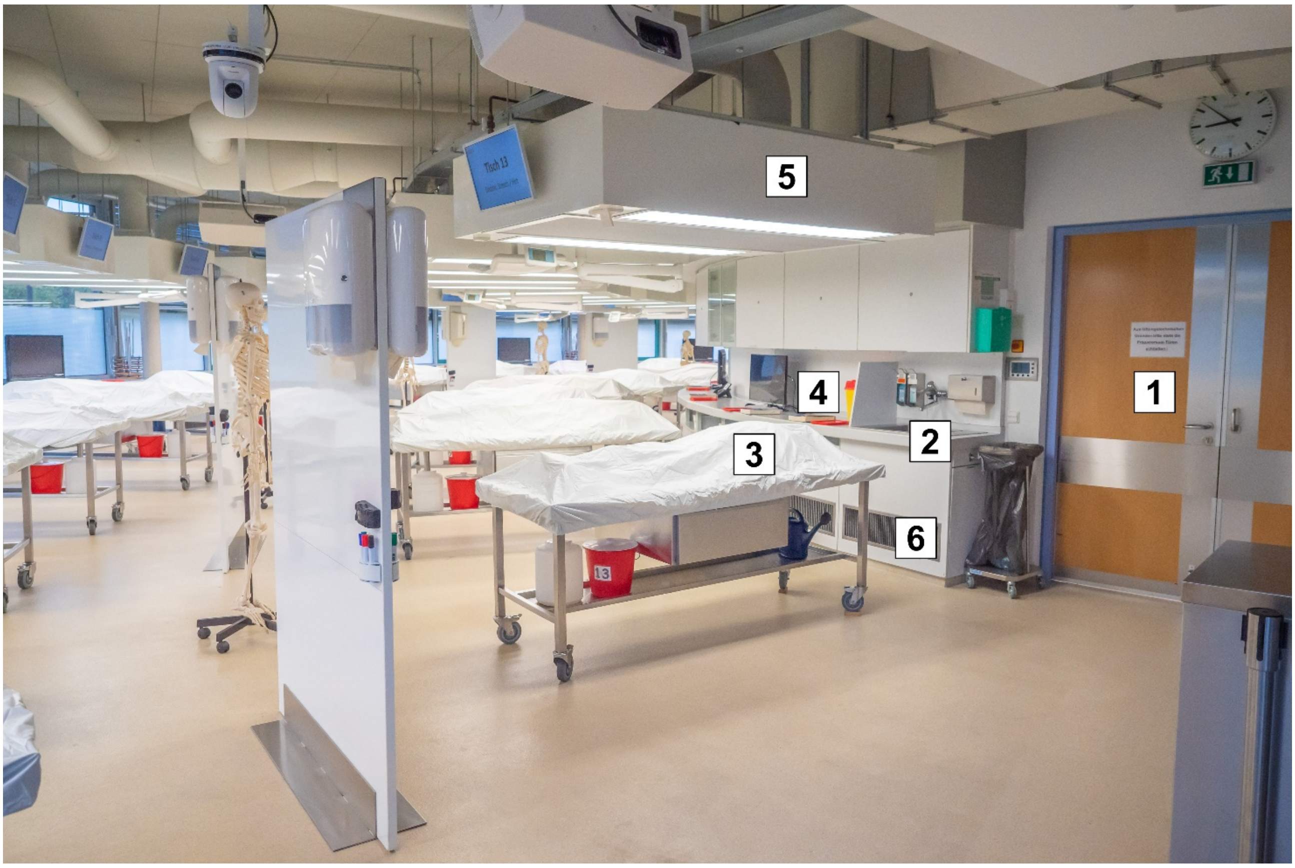

Supplementary Figure S3: Setting inside the dissection hall. 1: Inner side of the door providing access to the dissection hall. 2: Sink where students washed their instruments after dissection. 3: Dissection table no. 13, where sedimentation plate samples were acquired. 4: Sill next to dissection table 13, where sedimentation plate samples were acquired. 5: Laminar flow hood above the dissection table. 6: Waste air extract vent.

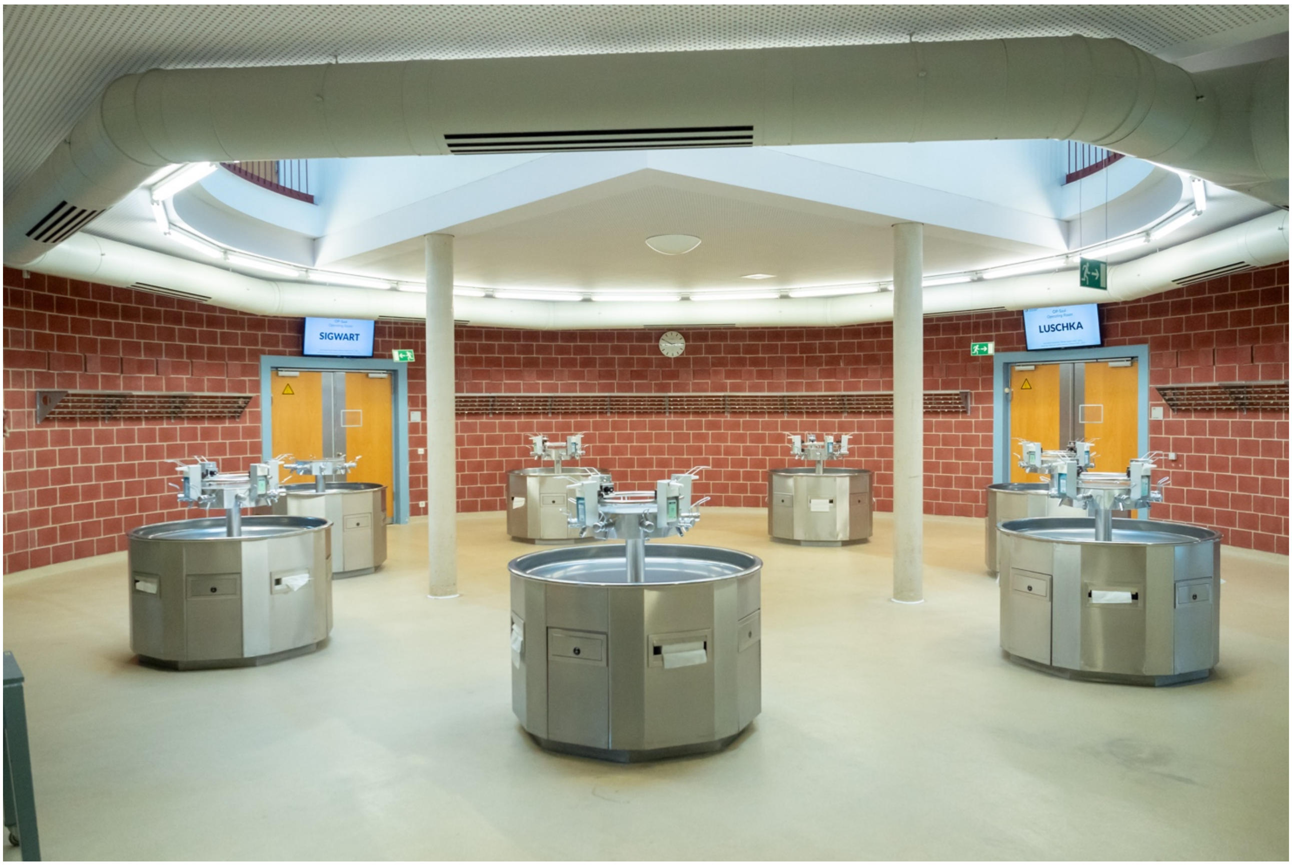

Supplementary Figure S4: Vestibule of the dissection hall, where students washed and disinfected their hands, and stored their gowns in between course days.

Supplementary Tables

Supplementary Table S1: Course schedule of the dissection course in winter term 2023/24. §: Acquisition of sedimentation and contact plate samples.

Course day	Date	Sample acquisition	Comments
0	16 October 2023	§	Baseline measurement without students inside the dissection hall
1	17 October 2023		
2	18 October 2023	§	
3	20 October 2023		
4	23 October 2023		
5	25 October 2023	§	
6	27 October 2023		
7	30 October 2023		
Exam preparation	2 November 2023		
8	3 November 2023	§	
Oral exam no. 1	6 November 2023		
9	8 November 2023		
10	10 November 2023		
11	13 November 2023	§	
12	15 November 2023		
13	17 November 2023		
14	20 November 2023	§	
15	22 November 2023		
Exam preparation	24 November 2023		
Oral exam no. 2	27 November 2023		
16	29 November 2023		
17	1 December 2023	§	
18	4 December 2023		
19	6 December 2023		
Exam preparation	8 December 2023		
20	11 December 2023	§	
21	13 December 2023		
Exam preparation	15 December 2023		
Oral exam no. 3	18 December 2023		

Supplementary Table S2: Airborne microbial load after exposure of the sedimentation plates for 4 hours, indicated as colony-forming units (CFUs) at dissection table 10, dissection table 13 and dissection table 16 and the sills next to the dissection tables. The sill next to table 13 (§§) is located directly next to a sink and the entrance of the dissection hall. On day 14, one sample (‡) had to be excluded due to touching the settle plate by hand by one of the students. A lawn growth (§) was observed at a total of five samples. The table represents the original data of Figure 3. CFUs: colony forming units. CNS: coagulase-negative staphylococci. MIC: micrococci. MND: molds (not differentiated). ASF: aerobic spore formers.

Day	Dissection table 10					Sill next to table 10					Dissection table 13					Sill next to table 13 (§§)					Dissection table 16					Sill next to table 16				
	CNS	MIC	MND	ASF	Total	CNS	MIC	MND	ASF	Total	CNS	MIC	MND	ASF	Total	CNS	MIC	MND	ASF	Total	CNS	MIC	MND	ASF	Total	CNS	MIC	MND	ASF	Total
**0**	0	0	0	0	0	0	1	1	1	3	0	1	1	1	3	0	1	2	2	5	1	0	0	0	1	1	0	0	1	2
**2**	2	0	0	2	4	21	23	2	9	45	8	6	0	2	16	22	40	9	200 (§)	272	7	4	5	2	18	3	21	14	10	48
**5**	4	1	0	1	6	12	10	3	9	34	5	3	0	2	10	20	24	10	9	63	2	1	1	0	4	10	13	0	15	38
**8**	14	11	0	0	25	32	17	0	15	64	8	3	2	4	17	0	0	200 (§)	0	200	10	3	3	3	19	21	6	2	7	36
**11**	10	9	0	10	29	40	24	1	10	75	12	8	0	3	23	42	87	1	27	157	3	3	0	5	11	19	24	0	8	51
**14**	‡	‡	‡	‡	‡	19	20	4	23	66	11	20	1	200 (§)	232	22	25	1	21	69	3	3	1	10	17	9	8	12	5	34
**17**	12	7	0	8	27	13	20	6	8	47	14	22	1	7	44	25	50	1	12	88	10	4	8	0	22	12	15	10	14	51
**20**	5	0	0	7	12	38	18	1	3	60	34	14	0	9	57	71	35	0	200 (§)	306	0	1	200 (§)	0	201	12	8	0	27	47
**Total**	47	28	0	28	103	175	133	18	78	394	92	77	5	228	402	202	262	224	471	1160	36	19	218	20	293	87	95	38	87	307

Supplemental Table S3: Airborne microbial load after exposure of the sedimentation plates for 1 hour, indicated as colony-forming units (CFUs) at dissection table 10, dissection table 13 and dissection table 16 and the sills next to the dissection tables. The sill next to table 13 (§§) is located directly next to a sink and the entrance of the dissection hall. A lawn growth (§) was observed at a total of two samples. CFUs: colony forming units. CNS: coagulase-negative staphylococci. MIC: micrococci. MND: molds (not differentiated). ASF: aerobic spore formers.

Day	Dissection table 10					Sill next to table 10					Dissection table 13					Sill next to table 13 (§§)					Dissection table 16					Sill next to table 16				
	CNS	MIC	MND	ASF	Total	CNS	MIC	MND	ASF	Total	CNS	MIC	MND	ASF	Total	CNS	MIC	MND	ASF	Total	CNS	MIC	MND	ASF	Total	CNS	MIC	MND	ASF	Total
**2**	2	3	0	0	5	5	9	0	3	17	1	4	0	2	7	8	26	2	5	41	4	3	0	1	8	6	8	1	2	17
**5**	1	1	0	0	2	5	3	0	5	13	1	0	0	0	1	13	17	0	5	35	1	0	0	0	1	8	2	0	2	12
**8**	5	1	0	6	12	3	1	0	2	16	3	1	0	0	4	0	0	200 (§)	0	200	4	2	0	2	8	0	1	1	0	2
**11**	0	7	0	1	8	10	5	0	3	18	7	6	1	0	14	16	32	10	0	58	3	2	0	0	5	12	13	0	4	29
**14**	0	3	2	1	6	3	8	1	5	17	15	12	3	6	36	20	30	0	14	64	0	0	3	0	3	0	5	1	200 (§)	206
**17**	4	5	1	3	13	9	6	0	2	17	7	10	0	4	21	14	44	0	15	73	2	3	0	1	6	7	10	0	4	21
**20**	2	0	0	7	9	12	10	0	3	25	8	3	0	3	14	16	22	1	2	41	0	1	0	4	5	2	3	1	1	7
**Total**	14	20	3	18	55	47	42	1	23	113	42	36	4	15	97	87	171	213	41	512	14	11	3	8	36	35	42	4	213	294

Supplementary Table S4: Microbial load, indicated as colony-forming units (CFUs) of the doorknob over the dissection course, before and after the respective course day. CNS: coagulase-negative staphylococci. The table shows the original data of Figure 4C+D. MIC: micrococci. MND: molds (not differentiated). ASF: aerobic spore formers.

Day		CNS	MIC	MND	ASF	Total
**2**	**before**	4	4	2	1	11
	**after**	24	9	0	2	35
**5**	**before**	0	0	0	0	0
	**after**	3	1	0	1	5
**8**	**before**	21	2	0	13	36
	**after**	7	3	0	5	15
**14**	**before**	14	15	0	15	44
	**after**	17	46	0	22	85
**17**	**before**	20	4	0	4	28
	**after**	10	3	0	0	13
**20**	**before**	25	2	0	9	36
	**after**	60	17	0	0	77
**Total**		**205**	**106**	**2**	**72**	**385**

Supplementary Table S5: Microbial load of the doorknob before and after the dissection course, pooled over all measurement timepoints. CFU: colony forming units. SD: standard deviation. CNS: coagulase-negative staphylococci. MIC: micrococci. MND: molds (not differentiated). ASF: aerobic spore formers.

	CNS	MIC	MND	ASF	Total
**Before [mean CFU ± SD]**	14,0 ± 10,0	4,5 ± 5,4	0,3 ± 0,8	7,0 ± 6,3	25,8 ± 16,9
**After [mean CFU ± SD]**	20,2 ± 20,9	13,2 ± 17,1	0,0 ± 0,0	5,0 ± 8,5	38,3 ± 34,6

Supplementary Table S6: Microbial load, indicated as colony forming units (CFUs), of the students' gown sleeves of three dissection tables. A lawn growth (§) was observed on one sample. The table shows the original data of Figure 5B+C. CNS: coagulase-negative staphylococci. MIC: micrococci. MND: molds (not differentiated). ASF: aerobic spore formers.

Day	Dissection table 10					Dissection table 13					Dissection table 16				
	CNS	MIC	MND	ASF	Total	CNS	MIC	MND	ASF	Total	CNS	MIC	MND	ASF	Total
**2**	1	26	0	6	33	6	3	0	200 (§)	209	3	0	0	16	19
**5**	3	10	1	4	18	6	4	0	13	23	5	3	0	3	11
**8**	16	19	0	9	44	6	5	0	4	15	34	5	2	19	60
**11**	17	0	0	5	22	17	9	0	6	32	1	1	0	1	3
**14**	23	6	1	7	37	41	6	0	20	67	3	7	0	16	26
**17**	3	0	0	30	33	21	31	0	18	70	9	4	0	6	19
**20**	14	1	0	3	18	48	0	0	0	48	18	0	0	3	21
**Total**	60	62	2	59	183	128	49	0	255	432	72	19	2	63	156

Supplementary Table S7: Information about the body donors in the dissection course in winter term 2023/24. #: Dissection table number. F: female, M: male. N/A: not available.

#	Sex	Age [y]	Body weight [kg]	Body height [cm]	Postmortal interval [h]	Storage after fixation [days]	Cause of death
1	F	79	85	160	62	498	Cardiac arrest
2	M	87	70	175	N/A	550	Gastrointestinal bleeding
3	F	72	44	168	N/A	495	Respiratory insufficiency due to tumor disease
4	M	86	80	170	34	242	Chronic kidney disease
5	F	85	48	160	38	456	Pulmonary edema
6	M	83	70	174	N/A	495	Pneumogenic sepsis
7	F	91	55	160	19	454	Aspiration pneumonia
8	M	82	89	175	N/A	466	Aspiration
9	F	88	N/A	N/A	N/A	431	Unknown
10	M	78	80	170	53	281	Multi organ failure
11	F	76	75	165	N/A	420	Respiratory insufficiency
12	M	80	60	170	19	414	Cardiopulmonary arrest
13	F	86	65	165	N/A	417	Pneumogenic sepsis
14	M	74	80	185	24	378	Pneumonia, cardiac failure
15	F	90	55	162	48	393	Cardiac failure
16	M	85	60	183	32	375	Chronic kidney disease
17	F	94	65	165	28	357	Marasmus
18	M	77	70	175	26	329	Multi organ failure
